# In vitro and in vivo neutralization of Dengue virus by a single domain antibody

**DOI:** 10.1093/immhor/vlaf012

**Published:** 2025-04-03

**Authors:** Surbhi Dahiya, Sudhakar Singh, Gaurav Kumar Bhati, Sharvan Sehrawat

**Affiliations:** Department of Biological Sciences, Indian Institute of Science Education and Research, Manauli, Punjab, India; Department of Biological Sciences, Indian Institute of Science Education and Research, Manauli, Punjab, India; Department of Biological Sciences, Indian Institute of Science Education and Research, Manauli, Punjab, India; Department of Biological Sciences, Indian Institute of Science Education and Research, Manauli, Punjab, India

**Keywords:** antibody dependent enhancement, Dengue virus, nanobodies, neutralizing single domain antibodies

## Abstract

To alleviate the contribution of antibody dependent enhancement in DenV pathogenesis, we obtain a DenV neutralizing single domain antibody (sdAb) from an in-house constructed phage display library of camelid V_H_H. The anti-DenV sdAb specifically reacts with the envelope (E) protein of DenV with a Kd value of 2x10^8^. Molecular dynamic simulations and docking analysis show that the sdAb interacts with the DenV(E) protein via domain II (EDII) and interferes with the virus internalization process. The anti-DenV(E) sdAb potently inhibits the infectivity of a DenV(E) protein expressing pseudovirus as well as that of a virulent DenV *in vitro*. A mouse adapted DenV2 induces 100% mortality in the infected IFNRKO mice, but the animals injected with the sdAb neutralized virus remain fully protected. Furthermore, the therapeutically administered anti-DenV(E) sdAb slows down the disease progression and enhances the survival of DenV infected animals. In conclusion, we report an anti-DenV(E) sdAb as a potential therapy to manage DenV pathogenesis.

## Introduction

Dengue virus (DenV) is transmitted by *Aedes aegypti* and the virus has become endemic in more than 120 countries. This makes half of the world's population prone to contract DenV infection. Residents of urban and semi-urban areas in tropical and sub-tropical countries are the most affected group.^1^ DenV exists in 4 serotypes, that is, DenV1 to DenV4, and the infected host exhibits a wide range of symptoms from a mild flu-like disease to severe and potentially life-threatening conditions. Primary infection with any of the serotypes and secondary infection with homologous serotype are usually resolved favorably. However, the secondary infection with a heterologous serotype can result in severe disease manifested as dengue haemorrhagic fever (DHF) and dengue shock syndrome (DSS).^2^ A phenomenon known as antibody dependant enhancement of infection (ADE) is primarily responsible for the DHF or DSS.^3^ Antibodies induced against 1 serotype can neutralize the homologous but not the heterologous serotypes. DenV complexed with such antibodies is preferentially internalized by immune cells such as monocytes and macrophages, which abundantly express Fc receptors.^4^^,^^5^ This not only enhances the infectivity but also induces a hyperinflammatory response. Therefore, developing effective vaccines against DenV has been challenging as the induced immunity provides inadequate protection to the host. Several platform technologies have been evaluated for developing anti-DenV vaccines which include live attenuated virus vaccine, chimeric live attenuated virus vaccine, inactivated virus vaccine, recombinant protein vaccine, and plasmid vaccine.^6^ Several vaccine candidates are currently undergoing clinical trials but the only approved vaccines are Dengvaxia and Qdenga which are restricted in several countries and these are not recommended for all the age groups.^7^ Therefore, there is an urgent need to develop better therapeutics to manage DenV disease.

DenV encodes 3 structural proteins (C, prM/M, and E) and 7 non-structural proteins (NS1, NS2A, NS2B, NS3, NS4A, NS4B, and NS5), each playing critical roles in viral assembly, replication, and immune evasion. The Envelope (E) protein is a surface expressed structural protein of the virus and is responsible for mediating viral attachment, fusion, and internalization. It consists of 3 domains viz., EDI, EDII, and EDIII. EDI, a central domain, is involved in the interaction of virus with the cellular receptor/s, while EDII is responsible for the dimerization of the protein, a process that facilitates the fusion of the virus with the cellular membranes. EDIII, an immunoglobulin-like domain, contains the receptor binding domain of the virus.^8–10^ Host cell surface receptors such as Dendritic Cell-Specific Intracellular adhesion moleculs-3-Grabbing Non-integrin (DC-SIGN), langerin, T cell immunoglobulin mucin domain protein (TIM) 1, 3, or 4 in addition to Tyro3, Axl, Mer (TAM), CD300a, Protein disulphide isomerase (PDI), CD14, FcγR by interacting with the viral protein to initiate the infection cycle.^11^ Several of these receptors are expressed by immune cells and therefore the recognition of viral proteins by such receptors can elicit immunopathological reactions. Given its critical role in the viral infectivity, DenV(E) protein is pursued as the primary target for anti-viral maneuvers.^12^^,^^13^

We selected a single domain antibody (sdAb) from phage display library of camelid V_H_H against the envelope protein of DenV and assessed its neutralization potential *in vitro* and *in vivo*. We show that anti-DenV(E) sdAb effectively neutralizes the infectivity of DenV. Due to the absence of Fc region, such antibodies are unlikely to cause ADE and hence can serve as a valuable therapy for managing DenV infection and the ensuing pathogenesis.^14^

## Materials and methods

### Mice, viruses, and cells

IFNRKO (B6.Cg-Ifngr1tm1Agt Ifnar1tm1.2Ees/J; Stock no. 029098) mice were obtained from Jackson laboratory, USA. The animals were housed, bred, and maintained in individual ventilated caging system at the Small Animal Facility for Experimentation (SAFE) of IISER Mohali. The animal experiments were performed in accordance with the approved protocols by the institutional animal ethics committee (IAEC). All the serotypes of DenV (DenV1, 2, 3, and 4) were obtained from Prof Guey Chuen Perng of National Cheng Kung University, Tainan, Taiwan. A mouse adapted strain of DenV2 was generated by infecting IFNR KO mice with 1,000 pfu of DenV2 through intraperitoneal injection. At 3 dpi, the collected organs such as spleen, lungs, liver, lymph nodes, and brain from the infected mice were homogenised in 1 ml of serum free DMEM using a Qiagen homogenizer, and the tissue homogenates were centrifuged at 13,000 rpm for 10 min to collect the supernatants. 100 µl of supernatants were injected into the fresh batch of IFNRKO mice, and these steps were repeated 8 times. The animals injected with the final passage of the virus developed signs of disease that were characterized by reduced body weight, hunched back posture, compromised mobility, and hypothermia. The animals that lost ∼20% of body weights were scarified to harvest different organs for analysis but for some experiments the survival was considered as the end point. The pooled supernatants were concentrated by mixing with 1.2M NaCl and 40% of PEG-8000 on ice for 3 to 4 h. The concentrated virus particles were collected by centrifugation at 3,500 g for 85 min, and the obtained pellet was resuspended in serum free DMEM. For some experiments, the other 3 serotypes DenV1, 3, and 4 were used after propagating in Vero cells. The virus was titrated and stored in small aliquots at −80°C for further use. The BMDCs were generated in 10% RPMI (10% FBS, and 10% Pen-strep). The HEK293T and Vero E6 cells were maintained in 10% DMEM.

### Cloning, expression, and purification of DenV(E) protein

DenV was propagated and titrated using Vero E6 cells. The virus was stored in small aliquots at −80°C. The viral RNA was isolated and converted to cDNA using verso cDNA kit as described earlier.^15^ Using specific forward and reverse primers, the genetic fragment for DenV (E) protein was amplified using cDNA as template (Table S1). The amplicon and the pMD2G vector were digested using PmlI and NotI restriction enzymes and extracted after resolving using agarose gels. The ligation of the PCR product and the vector was performed in 3:1 ratio at 25 °C for 2 and half h followed by incubating at 16 °C for 16 h. Stbl3 cells were transformed with the ligation mixture and the obtained colonies were screened using colony polymerase chain reaction (PCR) with the screening primers (T7 forward and T7 reverse; Table S1). The insert carrying clones were sequenced.

To express DenV(E) protein, HEK293T cells were seeded and maintained in 90 mm petri dishes in a humidified CO_2_ incubator at 37 °C. The cells at 70% confluency were transfected with 10 µg of DenV (E) plasmid pMD2G(E) in 30 µg of polyethyleneimine (PEI). pLenti-GFP served as an indicator of the transfection efficiency. The transfection mixture was vortexed for 40 s and then kept at 25 °C for 15 min. Complete DMEM was added to the cells after 6 h of transfection. The supernatants were collected 48 hr later and concentrated using 1.2M NaCl and 40% (W/V) PEG-8000 on ice for 3 to 4 h, which was followed by centrifugation at 3,500 g for 85 min. The precipitated pellets containing the DenV(E) protein were dissolved in 1X-PBS and stored at 4 °C. The concentrated protein was fractionated by size exclusion chromatography using S200 column, and the purification was assessed by SDS PAGE. The purified DenV(E) was used for biopanning and establishing the specificity of the selected antibody.

### Selection of anti-DenV sdAb using biopanning from the phage display library

An in-house constructed phage display library of camelid V_H_H was probed for selecting sdAb against the plate coated purified DenV(E) protein.^16^ TG1 bacterial cells were cultured overnight in 2X Yeast Tryptone (YT) medium supplemented with glucose. At 0.4 OD_600_ value of the secondary culture, the cells were infected with M13KO7 bacteriophage and kept in static condition at 30 °C for 40 min. Thereafter, kanamycin (50 µg/ml) was added and the cultures were kept overnight at 37 °C in a shaking incubator set at 200 rpm for the multiplication of bacteriophages. The following day, the cultures were centrifuged at 5,000 rpm for 20 min at 4 °C, and the collected supernatants were precipitated using 20% PEG and 0.5M NaCl on ice for 1 h. The precipitates were pelleted by centrifugation at 5,000 rpm for 30 min. The pellets thus obtained were resuspended in 1XPBS. TG1 bacterial cells were infected with the propagated helper phages and transformed with phagemid containing V_H_H sequences for packaging and subsequent release of the infectious bacteriophages.

Purified DenV(E) protein (10 µg/ml) was coated onto ELISA plates overnight at 4 °C. The plates were then washed with freshly prepared PBS-1% Tween-20 (PBST) and blocked with 4% BSA for 2 h at room temperature (RT). The wells were washed thrice with PBST and subsequently added with 10^12^ of recombinant phages/well for 3 hrs at RT. More than 30 washings were done to remove unbound or loosely interacting bacteriophages. High affinity binders were eluted using freshly prepared alkaline triethylamine acetate (TEA) buffer. The eluted phages were then enriched by performing second and third rounds of biopanning.

### Cloning and expression of anti-DenV(E) sdAb

TG1 bacterial colonies were screened using vector specific forward primer, MKSS9 and the insert specific reverse primer, MKSS22 (Table S1). V_H_H sequences were subcloned into pYBNT (modified pET22b^+^) vector which was then transformed into Origami cells. Colony PCR was performed to confirm the V_H_H construct using T7 forward and reverse primers. Randomly chosen colonies were induced with 1 mM IPTG in 5 ml of LB broth supplemented with 100 µg/ml of ampicillin. The sdAb expressing clones were sequenced and propagated further for a large scale production for which a primary culture of 10 ml was propagated overnight in LB broth supplemented with ampicillin (100 µg/ml) at 37 °C. One liter of secondary culture grown at 0.4 to 0.6 OD_600_ values was induced with 1 mM of IPTG at 37 °C for 5 h. Bacterial pellets obtained by centrifugation at 8,000 rpm at 4 °C for 15 min was used to purify the sdAbs.

### Purification of sdAb from inclusion bodies

The expressed sdAb predominantly accumulated as inclusion bodies in *E. coli.*^17^ The bacterial pellet was first resuspended in lysis buffer containing 100 mM Tris base and 10 mM EDTA. The bacterial suspensions were sonicated at an amplitude of 25 with eight cycles each of 59 seconds on and 59 seconds off while keeping the bacterial cells on ice. Subsequently, the cells were centrifuged at 8,000 rpm for 15 min at 4 °C, the pellets were washed twice with wash buffer A (100 mM Tris base, 10Mm EDTA, 1M NaCl, pH 8.0) and twice with wash buffer B (100 mM Tris Base, 10Mm EDTA, 1% v/v Triton 100; pH 8.0). The pellets obtained were finally resuspended in denaturation buffer containing 100 mM NaH_2_PO_4_, 10 mM Tris-HCl, 8 mM urea, pH 8.0 by rotation for 18 to 20 h at 4 °C. The denatured fractions were centrifuged at 5,000 rpm at 4 °C for 20 minutes to obtain clear extracts. The sdAb was affinity purified using Ni-NTA and HIS-trap columns pre-equilibrated with the denaturation buffer. The non-specific interactors were washed with 20 mM imidazole in denaturation buffer (pH 8.0). The bound product was eluted using 400 mM imidazole in denaturation buffer (pH 7.8). The protein thus obtained was mixed with an equal volume of guanidine solution (3M Guanidine hydeochloride (GuHCl), 10 mM sodium acetate and 10 mM EDTA, pH 4.2) and was refolded following a rapid dilution method in the refolding buffer (100 mM Tris, 1 mM EDTA, 1 mM reduced glutathione (GSH), 0.1 mM oxidised glutathione (GSSG), 400 mM arginine) as described earlier.^18^^,^^19^ The refolded fraction was then subjected to size exclusion chromatography using an S200 Hi-prep column (GE Healthcare, USA). The refolded sdAb was concentrated using 3 kDa cutoff concentrator (Amicon) to 1 ml volume. The concentrated sdAb preparation was also resolved using size exclusion S200 column chromatography to determine the elution characteristics and homogeneity of the purified product.

### Refolding of the sdAb and determining its specificity

#### Refolding characteristics of the sdAb

Concentrated samples of DenV(E) and anti-DenV sdAb were analysed by circular dichroism (CD) to evaluate secondary structures.

#### Immunological reactivity by ELISA

ELISA plates were coated with 5 µg/ml of purified DenV(E) protein in 100 µl volume/well overnight at 4 °C. The wells were then washed with 0.05% PBST and blocked in 5% BSA in PBST for 2 h at RT. Anti-DenV(E) sdAb was added in graded concentration (0, 10, 20, 30, 40, and 50 µg/ml) for 1.5 h at RT. For detection of sdAb, streptavidin-HRP was incubated in 1:1000 dilution for 1 hr at RT. Thereafter, plates were washed thrice with PBST and developed using TMB substrate. 50 µl of stop solution (1 N ortho-phosphoric acid) was added after developing the plates and absorbance was measured at 450 nm. Additionally, the specificity of anti-DenV(E) sdAb was established against the whole virus. Graded concentrations of the inactivated DenV2 (0, 10^2^, 10^4^,10^6^ pfu) were coated onto the ELISA plates overnight at 4°C. The plates were washed and blocked with 5% BSA in PBST for 2 h at RT. Thereafter, the plates were added with 20 µg/ml of either monomeric or the tetramerized sdAb for 2 h at RT. The sdAb was detected with streptavidin HRP using TMB as the substrate for developing plates. For performing competitive ELISA, the plates were coated with live inactivated DenV (10^4^pfu/ml) overnight at 4°C followed by blocking with 5% BSA. 20 µg/ml of the anti-DenV sdAb preincubated for 90 min at 4°C with the graded concentration (0, 0.1, 1, 10, 20, 40 µg/ml) of DenV(E) protein were added. The plates were kept at RT for two hrs and thereafter the plates were developed using streptavidin-HRP as described above.

#### Measuring the affinity of interaction using biolayer interferometry (BLI)

To determine the binding kinetics of anti-DenV(E) sdAb with the purified recombinant DenV(E) protein produced in the transfected HEK293T cells, BLI was performed using BLltz System. Ni-NTA probes (ForteBio) were used to immobilise 6x(HIS)-tagged anti-DenV(E) sdAb. In total, 100 µl of sdAb (300 μg/ml) was loaded for 5 minutes which followed washing with PBS to remove unbound protein. Different concentrations of DenV(E) protein were incubated for 5 min with the immobilised anti-DenV(E) sdAb to measure the binding affinity by calculating the dissociation constants. Recharging of the probes was done by placing the sensor in 10 mM glycine for 1 min and then in PBS for 5 minutes. Finally, the probes were placed in 10 mM Nickel sulphate solution for 1 min.

### Western blotting

DenV(E) protein purified from the transfected HEK293T cells or the LV (DenV-E) pseudoviruses, were resolved using 12% SDS-PAGE and the resolved polypeptides were transferred to PVDF membranes. The membranes were blocked with 5% skimmed milk for 12 to 14 h at 4°C. To the blocked membrane, DenV(E) sdAb containing 6x(HIS)-tag was added for detection. Mouse anti-6x(HIS) antibody (4A12E6: Invitrogen Rockford, USA) was used to detect anti-DenV(E) sdAb. Finally, a goat anti-mouse IgG antibody (A3562: Sigma, USA) conjugated with alkaline phosphatase was added and thereafter the membranes were developed.

Lentivirus based pseudoviruses expressing DenV envelope protein; LV(DenV-E) and bald particles; LV(BALD) were resolved using 12% SDS-PAGE and then transferred onto PVDF membrane. Blocking of the membranes was done with 5% skimmed milk followed by probing with biotinylated sdAb for 1.5 h at RT. The membrane was further incubated with streptavidin HRP (life technology, SNN1004) (1: 50,000 dilutions) for detection of the biotinylated sdAb. The membranes were developed using ECL substrate (BioRad).

To assess the ability of anti-DenV(E) sdAb in hampering the virus internalization, DenV particles (10,000 pfu) were pre-incubated with 40 µg of sdAb for 1.5 h at 4°C, and the mix was later overlaid on Vero E6 cells in 6-well plates for 3 d. Thereafter, the DenV infected control cells or those incubated with the virus pre-neutralized by anti-DenV(E) sdAb were collected to prepare cell lysates for probing by western blotting using the biotinylated anti-DenV(E) sdAb as the primary antibody and streptavidin-HRP as the conjugate for developing the membranes.

### Molecular docking and molecular dynamic simulation

The interaction of anti-DenV(E) sdAb with the DenV(E) protein was assessed using Colab alphaFold 2 by docking the predicted structures of the antibody and the envelope protein (PDB_10AN) using a docking server ClusPro (https://cluspro.org). The most stable model obtained from ClusPro was used for performing MD simulation using the QwikMD plugin into VMD and the simulations were run with NAMD version 2.12 using the CHARMM36 force field.^25^^,^^26–30^ The model structures were aligned with the longest axis along the *Z*-axis. The system was solvated using a water box of the dimensions 86.35, 122.36, 176.68  Å in X, Y, and Z directions placed at (−45.13, −60.49, −88.67) and (41.22, 61.87, 88.01) as the minimum and maximum coordinates. Transferable intermolecular potential 3P (TIP3P) water was used for the solvation. The molecule was also rotated to minimize the solvent box volume. Na^+^ and Cl^−^ ions corresponding to a concentration of 150 mM were then placed randomly in the water box by replacing the water molecules. The system was then minimized for 5,000 steps with the position of the protein atoms fixed which was followed by 5,000 steps without any restraints. A distance cutoff of 12  Å was applied to short-range, non-bonded interactions, and 10  Å for the smothering functions. The temperature of the system was then gradually raised to 300K at a rate of 1K every 600 steps with the backbone atoms restrained. This was followed by equilibration for 5 ns with the backbone atoms restrained. Thereafter a 100 ns production simulation run was performed. For all the steps, the pressure was maintained at one atm using the Nose-Hoover method, the long-range interactions were treated using the particle-mesh Ewald (PME) method, and the equations of motion were integrated using the r-RESPA scheme to update the short-range van der Waals interactions for every step and the long-range electrostatic interactions every two steps.^28^^,^^31–33^

Root-mean-square deviation (RMSD) measures the structural distance between coordinates and the average distance between a group of atoms. It was calculated using the following equation from molecular dynamics trajectory.


$$\mathrm{RMSD}=\;\sqrt{\frac{\sum_{i}^{N}{\left(x_{i}-\;{\bar{x}}_{i}\right)}^{2}}{N}}$$

Where N is the number of residues whose position is being compared, $i^{\mathrm{th}}$ is the position of the residues in the reference frame. $\bar{x_{i}}$ is the average value of the $i^{\mathrm{th}}$ atom. RMSD value was measured by the abovementioned equation till 100 ns long simulation.

### Generation of LV(DenV-E) pseudovirus

Pseudotyped lentiviruses expressing surface DenV(E) protein were generated by transfecting HEK293T cells using the modified pMD2G plasmid that encoded DenV(E) protein, a packaging vector (pCMVR8.74), a pLenti-GFP (Core protein with 5′ and 3′ LTR) from Addgene (no. 17448) and the Tat1b and Rev1b plasmids obtained from BEI resources. All these regents and the methods were described earlier.^20^ Briefly, the DenV(E) gene sequence was inserted in the pMD2G vector using *PmlI* and *NotI* restriction sites. The plasmids viz., pMD2G-DenV(E) (6 μg), pLentiGFP (10.8 μg), pCMVR8.74 (9.8 μg), Tat plasmid (6 μg) and Rev plasmid (6 μg) were mixed with the transfection reagent, PEI (polyethleneimine) (1 mg/ml) in 1:3 ratio (DNA content: PEI in 1 ml of serum free DMEM for each petri-dish). The mix was then vortexed for 40 seconds and then kept at room temperature for 15 min before layering onto HEK239T cells. The transfected HEK293T cells were supplemented with freshly prepared 10% DMEM after 6 h of transfection. The replication incompetent pseudotyped lentivirus (LV) expressing (DenV) envelope protein; LV(DenV-E) and control particles displaying VSV-G surface protein; LV(VSV-G) were harvested from the culture supernatants after 72 h of transfection. The collected supernatants were centrifuged at 200×*g* to remove cellular debris. The supernatants were then concentrated using 40% (W/V) PEG-8000 and 1.2MNaCl on ice for 3 to 4 h followed by centrifugation at 3,500 g for 85 min. The pellets were dissolved in serum free medium, stored at 4**°C** for short or at −20**°C** for longer duration. The concentrated pseudoviruses were resolved by SDS-PAGE for determining the expression of DenV(E) protein.

### Measuring neutralization of pseudoviruses by anti-DenV(E) sdAbs

Vero E6 cells were used for assaying the internalisation of pseudoviruses and inhibition by the anti-DenV(E) sdAb. Vero E6 cells were cultured in complete DMEM supplemented with 10x penicillin/streptomycin and maintained in a humidified CO_2_ incubator at 37**°C**. Following the infection, Vero E6 cells were supplemented with complete DMEM after 24 h of infection. After 72 h, the cells were removed from the culture plates using 1 mM PBS-EDTA and GFP expression was measured using flow cytometry. To measure the effect of anti-DenV(E) sdAb in internalization of LV(DenV-E), the graded concentrations of the sdAb (1 ng, 10 ng, 100 ng, 500 ng, 1 µg, and 10 µg/ml) were pre-incubated with 5 × 10^6^ particles on ice for 90 mins. Thereafter, the mixtures were separately over layered onto 70% to 80% confluent Vero E6 cells. The infected cells were analysed for GFP expression 48 h later.

### Measuring in vitro neutralization of live replicating DenV virus by anti-DenV(E) sdAbs

For assessing *in-vitro* neutralization of DenV2 by anti-DenV(E) sdAbs, 10,000 pfu were pre-incubated with the increasing concentrations of anti-DenV(E) sdAbs (0 µg, 5 µg, 10 µg, and 40 µg) for 90 minutes on ice. The mixtures were then added to Vero E6 cells for 90 min at 37°C followed by replacing the supernatants with fresh medium. The virus titers in each group were measured by plaque forming assays.

### Measuring the effects of anti-DenV sdAb in interfering virus attachment

10,000 pfu of DenV2 was incubated on ice with the graded concentrations (0, 1, 10 and 50 µg/ml) of anti-DenV(E) sdAb for 90 min. The mixture was then added to Vero E6 cells for 1 h at 4°C. Supernatants containing the unattached viruses were discarded, and the cells were washed thrice with 1×PBS. Thereafter, the cells were harvested using 1 mM PBS-EDTA and surface stained with 10 µg/ml of biotinylated anti-DenV(E) sdAb in 50 µl volume. Percentage of cells showing positive staining were analysed by Flow cytometry.

### DenV infection of mice

IFNRKO mice were infected with different doses of DenV2 (1pfu, 10pfu, 100pfu) using intraperitoneal (i.p) route to calculate the infectious dose. Different parameters such as change in bodyweight, survival and disease scores were measured for animals in different groups. The infected mice were scored on a scale of 1 to 5 based on the signs and symptoms of disease. The following scoring scheme was followed: (1) apparently healthy animals, (2) mildly lethargic animals, (3) lethargic animals with ruffled fur and hunched back posture, (4) lethargic animals with ruffled fur, hunched posture, and reduced mobility, and (5) moribund animals.^21^

### In vivo neutralization of DenV with anti-DenV sdAb

Graded concentrations (0, 20, 40, 80 µg/ml) of anti-DenV(E) sdAb were pre-incubated with 1pfu of DenV2 for 90 min at 4°C. The mixture was then injected using i.p route in *IFNRKO* mice. Disease progression was monitored. Uninfected and the DenV2 infected animals without anti-DenV(E) sdAb therapy served were used as control. In separate experiments, IFNRKO mice infected with 1pfu of DenV were injected with 100 µg of anti-DenV(E) sdAb daily until the termination of experiments. The animals were monitored for the progression of DenV- induced disease. Separate groups of animals were similarly infected with the virus and the samples collected were analysed by qRT-PCR, confocal microscopy and H&E staining for assessing lung pathology. Additionally, to evaluate the toxicity induced by the anti-DenV sdAb, IFNKO mice were injected with 100 µg of anti-DenV sdAb, 1 dose per day for 4 d. Another group was infected with 1 PFU of DenV, while a third group was administered 100 µg of anti-DenV(E) sdAb a day post-infection. All groups were sacrificed at 5 dpi, and lung and spleen tissues were isolated for RNA extraction. The transcripts for the NS3 gene, along with pro-inflammatory cytokines and anti-inflammatory cytokines IL-4 and IL-1β, IL-10 and IL-6 were analyzed.

### qRT-PCR for determining the viral load

The abundance of DenV transcripts in tissues of the infected animals were determined through qRT-PCR. RNA isolated from both the infected and uninfected mice was converted into cDNA using verso cDNA kit. NS3 gene was amplified using the template cDNA with specific primers (Table S1). The Ct values were analysed using ^–ΔΔ^CT method.^22^ Also, 18sRNA was used as a housekeeping gene whose primer sequences are shown in Table S1.

### Antibody and other reagents

Cells of innate and adaptive immune system in the DenV infected animals were analyzed using antibodies obtained from BD biosciences, eBiosciences, Tonbo biosciences and BioLegend. The antibodies used were against CD11c-PE-Cy7/PE (N418), CD11b-FITC (M1/70), CD8-PerCP-Cy5.5 (530-6.7), Gr1, CD4, and F4/80. Anti-mouse-GAPDH (GA1R) along with anti-DenV NS1 Ab (SAB2702308) in addition to the anti-DenV(E) sdAb were used for western blotting. GM-CSF (315-03) and IL-4 (214-14) obtained from PEPROTECH were used for generating bone marrow derived dendritic cells (BMDCs).

### Differentiation of bone marrow cells and their DenV infection

The bone marrows were isolated to prepare single cell suspension. RBCs were lysed, and the cells were washed twice with PBS followed by resuspension in complete RMPI. The cells were then seeded in a 90 mm petri dish and supplemented with 5 ng/ml of the GM-CSF. On third day, the media was replaced with the fresh medium supplemented with 5 ng/ml of GM-CSF. . The generated BMDCs were scraped off the plates and neutrophils were collected from the suspension media on day 5. These cells were surface stained with anti-CD11b and anti-CD11c antibodies for determining the frequency of DCs and CD11b and Gr1 for those of neutrophils. Equal number of BMDCs and neutrophils were separately seeded in 96 well plates and pulsed with 0.5MOI of DenV for 2 h. After the infection, the cells were washed extensively with PBS and the percentage of infected cells was ascertained by intracellular staining with anti-DenV(E) sdAb. After establishing the infectivity, the infected cells were labelled with Cell Trance and then adoptively transferred into gender matched IFNRKO mice. Blood staining was performed the following day to determine the equal transfer of cells in all the animals and the mice were then monitored for the disease progression. The samples were acquired using BD C6 flow cytometer and analysed using FlowJo software.

### Flow cytometric analysis of cells

In order to perform surface staining of cells, different organs of mice were harvested in 10% RPMI to prepare single cell suspensions as described earlier.^23^ The cells were stained using the indicated antibodies by incubating on ice for 30 min. The cells were washed thrice by centrifugation at 200×*g* for 5 minutes in 1× PBS at 4°C and acquired using BD C6 flow cytometer.

For measuring intracellular DenV proteins, equal number of cells from different organs were surface stained, fixed and permeabilised using fix-perm buffer for 30 min on ice. The intracellular DenV antigens were probed using biotinylated anti-DenV(E) sdAb on ice. Anti-DenV(E) sdAb was detected using streptavidin-PE, and the cells were acquired using BD C6 flow cytometer.

The transfected HEK293T cells or the DenV2 infected Vero E6 cells after 72 h were treated with 1X PBS-1mM EDTA for 15 min at 37°C in CO_2_ incubator and were removed by gentle reverse pipetting. Cells were collected in 1.5 ml microcentrifuge tubes, washed twice with 1X PBS and acquired using flow cytometer (BD Accuri). The data was analysed using flowJo X software (TreeStar).

### Measuring viral loads in the organs of DenV infected animals

The viral load in different organs of the infected mice was measured using plaque assays. Tissue homogenates from organs such as spleens, lungs and brains were prepared in serum free DMEM with a tissue homogenizer. The homogenates were centrifuged at 13,000rpm for 10 min at 4°C and the collected supernatants were titrated for measuring the DenV loads. The plaques were developed and visualised by crystal violet staining as described earlier.^24^

### Fluorescent microscopy and histopathology

To assess the accumulation of DenV antigens in organs of the virus infected controls and anti-DenV sdAb injected animals, the animals were euthanised at 3 and 6 dpi. Different organs collected from the scarified animals were analysed for assessing the viral antigens as well as cellular infiltrates. Briefly, lung tissues from the different groups of animals were isolated and fixed using 4% paraformaldehyde overnight. Thereafter, the samples were dehydrated with a gradient of 5% to 20% sucrose solution at room temperature (RT) and embedded in OCT compound. 10 µm sections were cut using a cryotome and overlaid on lysine coated slides. Dried samples were first blocked with 4% BSA for 1 h at RT, washed thrice with PBS and probed with mouse anti-NS1 (DenV) antibody overnight at 4°C. After washing thrice with PBS, these sections were stained with a cocktail of Hoechst, anti-mouse IgG-Alexa Fluor 647 and Alexa Fluor^TM^ 488 phalloidin for 1 h at RT. The slides were washed thrice with 1XPBS and finally mounted with fluoromount-G. The slides were visualized using Nikon A1 confocal microscope and the images were analysed by using Image J software. Additionally, sections from lung tissues were stained using haematoxylin and eosin Y (H and E). Briefly, the tissue sections of lungs were air dried and kept in xylene (ready to use solution AS133) for 2 min and then hydrated in decreasing concentration of ethanol (absolute alcohol for 3 mins followed by 95% and 70% ethanol for 2 min each). The slides were then washed with tap water followed by staining with haematoxylin (TC259) for 8 min. The slides were again washed with tap water for 5 min to remove excessive stain and then stained with eosin for 1 min. The excessive stain of eosin (Himedia-S007) was removed by dehydrating with 95% ethanol for 1 min and then clearing with xylene for 1 min. The slides were then mounted with fluoromount-G and left at room temperature for 14 hr. The sections were visualized and the images were recorded using Leica DMi8 microscope.

### Statistical analysis

Statistical analysis was performed using GraphPad Prism9. The data were analyzed by 1-way ANOVA, 2-way ANOVA or non-parametric t tests as indicated. All the experiments were repeated at least 3 times. Data is represented as mean ± SEM values and the level of statistical significance was determined as ∗∗∗∗*P* < 0.0001, ∗∗∗*P* < 0.001, ∗_*_P < 0.01, and ∗*P* < 0.05.

## Results

### Selection and characterization of anti-DenV(E) sdAb from phage display library

**Figure 1.**
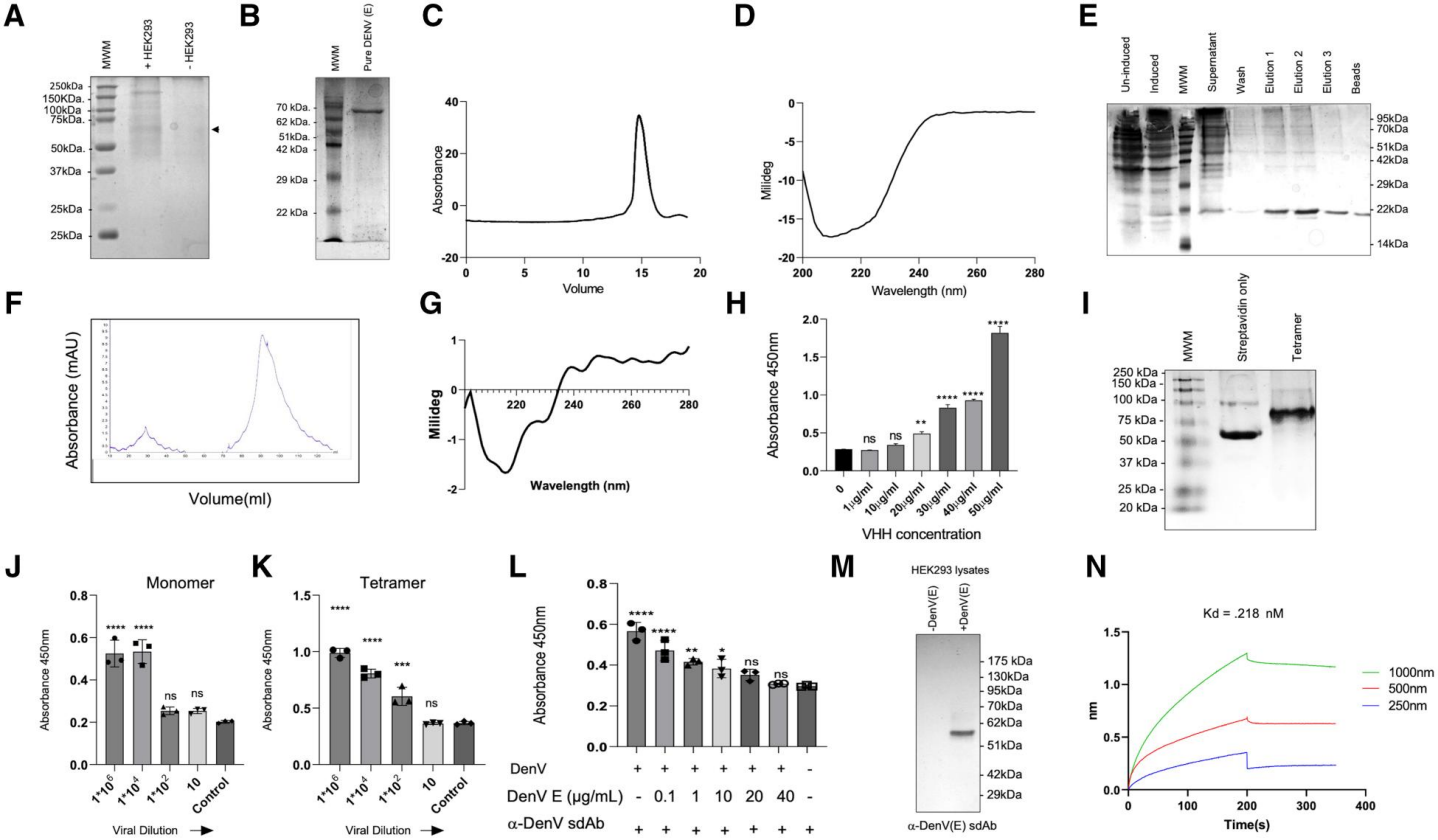
Purification of recombinant envelope protein, identification and characterisation of DenV envelope specific anti-DenV(E) sdAb. (A) HEK293T cells were transfected with the DenV(E) plasmid construct to produce the recombinant protein. After 72 hrs of transfection, the supernatant collected from the vector control and the DenV(E) transfected cells were resolved using 12% SDS PAGE. (B) The concentrated supernatants using PEG-NaCl were subjected to size exclusion chromatographic separation and the chromatogram is shown. (C) The fractionated envelope protein was assessed for purity by resolving using 12% SDS PAGE. (D) The refolding of the envelope protein was confirmed through circular dichroism. (E). The anti-DenV(E) sdAb was purified from the inclusion bodies and confirmed by resolving using 12% SDS PAGE. (F) Purified anti-DenV sdAb was refolded and isolated using size exclusion chromatography. (G) The refolding characteristics of the anti-DenV(E) sdAb were confirmed by circular dichroism. (H) The binding of anti-DenV(E) sdAb to the coated DenV(E) proteins was shown by the results of indirect ELISA where graded concentrations of anti-DenV (E) sdAb was used to probe the recombinant protein. Bar graph shows the result of indirect ELISA. (I) Biotinylated anti-DenV(E) sdAb was tetramerised using streptavidin. SDS-PAGE shows the tetrameric anti-DenV sdAb. (J) and (K) Bar graph shows the results of ELISA using the monomeric (J) and tetrameric version (K) of anti-DenV(E) sdAb used for detection of DenV at indicated dilutions. (L) The specificity of interaction of anti-DenV sdAb with envelope protein is shown by bar graphs wherein the varying concentrations of the soluble DenV(E) protein were added to 10µg/ml of anti-DenV(E) sdAb and the blocked antibody was used to detect plate bound DenV(E) protein using a competitive ELISA. (M) DenV(E) protein produced by the transfected HEK293T cells was probed using anti-DenV(E) sdAb by western blotting. (N) Line graph showing the binding affinity of anti-DenV(E) sdAb with the purified DenV(E) protein as determined using BLI (biolayer interferometry). The experiments were repeated at least three times and representative data from one of the experiment is shown. Mean ± SEM values are shown and the levels of statistical significance was analyzed by 1-way ANOVA. ****P < 0.0001; ***P < 0.001; **P < 0.01; *P < 0.05.

Envelope protein of DenV was expressed by transfecting HEK293T cells and a polypeptide band migrating at ∼60kDa was evident in the transfected cells (Fig. 1A). The protein was Ni-NTA purified from the culture supernatants. The concentrated protein was resolved using size exclusion chromatography and SDS-PAGE. We obtained a predominant polypeptide migrating at ∼60kDa range in the stained gels and a single prominent peak in the chromatogram (Fig. 1B and C). CD analysis of the purified DenV(E) protein showed the presence of a sharp dip near 208 nm and a minor peak near 222 nm that corresponded to predominantly alpha (α) -helices with beta (β) sheets in the structure (Fig. 1D). The protein was used as a bait for selecting sdAbs from the phage display library. More than 100 colonies were obtained after biopanning and multiple colonies were screened for the inserted V_H_H sequences. Out of 24 colonies, 10 were randomly chosen for sub cloning into pYBNT (modified pET22b^+^) vector and the cloning was confirmed using T7 forward and reverse primers. Several colonies were IPTG-induced at a small scale. Five clones expressing the V_H_H were sequenced and the one expressing high levels of sdAb was propagated further in one litre of the bacterial culture for the expression of V_H_H using 1 mM IPTG for 5 h at 37°C. The expressed recombinant protein was purified from inclusion bodies by first sonicating the pellet and then passing the bacterial supernatant through Ni-NTA beads (Fig. 1E). The purified anti-DenV(E) sdAb was refolded using a rapid dilution method and the refolded product was resolved using size exclusion chromatography (Fig. 1F). In addition to a major peak, a minor peak at the void volume was observed in the chromatogram representing protein aggregates (Fig. 1F). The concentrated fractions of the dominant peak were analysed by CD. We obtained a prominent peak nearing 218 nm and a minor peak at 230 nm indicating the presence of β-sheet and intrinsic di-sulphide bonds, respectively, in the structure of the sdAb (Fig. 1G).

### Establishing immune reactivity and binding affinity of anti-DenV(E) sdAb with DenV(E) protein

To measure the specificity of interaction between anti-DenV(E) sdAb and the viral E protein, several biophysical and immunological assays were performed. DenV(E) (5 µg/ml) protein was plate-coated and different concentrations of the sdAb were added. Absorbance at 450 nm revealed a concentration dependant increase in the binding of anti-DenV(E) sdAb with the immobilized DenV(E) protein (Fig. 1H). The anti-DenV(E) sdAb was biotinylated and tetramerised using streptavidin to increase its valency as well as the binding avidity of interaction with the DenV(E) protein. The anti-DenV(E) sdAb incubated with streptavidin revealed a slower migrating band of ∼100kDa as compared to that faster moving band of ∼50kDa (Fig. 1I). We then compared the binding of monomeric and the tetramerised anti-DenV(E) sdAb. While the monomeric version detected 10^4^ pfu/ml of the coated DenV particles its tetrameric version detected 10^2^ pfu/ml of the coated virus particles in ELISA (Fig. 1J and K). The anti-DenV(E) sdAb preincubated with varying concentrations of the purified DenV(E) protein when probed against the plate coated UV-inactivated DenV showed a concentration dependent reduction in the absorbance values illustrating its specificity of binding (Fig. 1L). We also determined the specificity of the interaction between anti-DenV(E) sdAb and DenV(E) protein by western blotting. The cell lysates prepared from the DenV(E) construct-transfected HEK293T cells were resolved using a 12% SDS-PAGE and the transferred polypeptides were probed with anti-DenV(E) sdAb. A bright intensity band migrating at ∼60kDa was clearly evident in the transfected cells but not in the control cells suggesting for a specific recognition (Fig. 1M). The affinity of interaction of anti-DenV sdAb with the DenV(E) protein as calculated by the dissociation constant (Kd) was 2.1 × 10^−8^ (M) (Fig. 1N). Therefore, we isolated a specific sdAb against the DenV(E) protein.

### Anti-DenV(E) sdAb neutralizes the infectivity of DenV in cell culture assays

**Figure 2.**
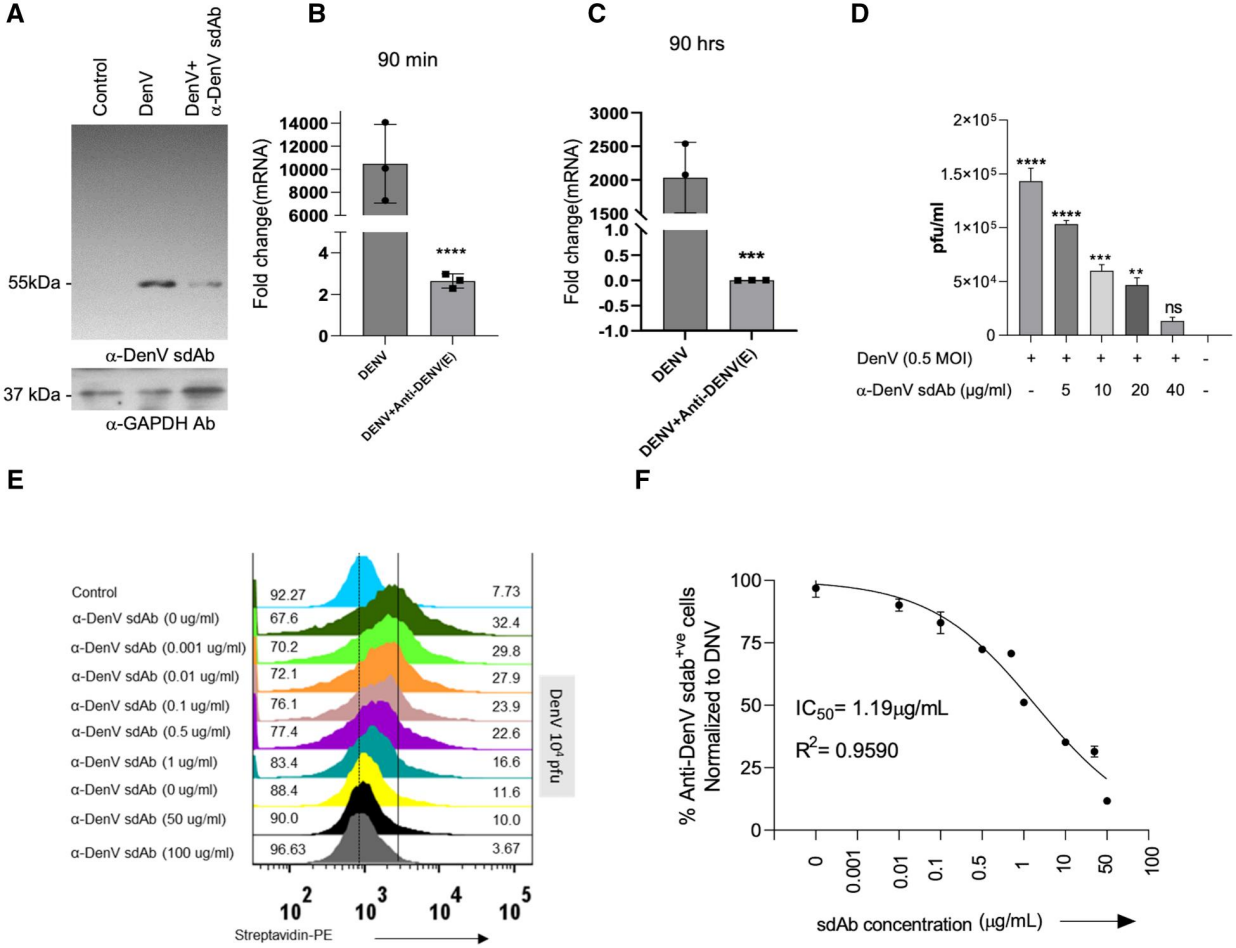
Measuring the neutralization DenV and LV(DenV-E) pseudoviruses using anti-DenV(E) sdAb. (A) Vero E6 cells were infected with either DenV or with a mixture of DenV and anti-DenV(E) sdAb pre-incubated for 1.5hrs. The infected cells were harvested to prepare lysates and the resolved polypeptides were probed with anti-DenV(E) sdAb. Immunoblots show the relative abundance of DenV(E) in the indicated condition. (B, C) qPCR results show the transcript abundance of NS3 of DenV in the infected Vero E6 cells at 90 minutes (B) and 90 mins post infection (C) 90 h post infection. (D) Graded doses of anti-DenV(E) sdAb were added to DenV for 90min at 4°C and then the mix was overlaid onto Vero E6 cells. After 3 d, the plaques were counted and shown by bar graph. (E) Representative offset histograms show the frequencies of Vero E6 cells scored positive for the surface attached DenV in the in the presence of varying concentrations (0, 0.001, 0.01, 0.1, 1, 10, 50,100 microgram/ml) of anti-DenV(E) sdAb. (F) Graph shows the calculated IC_50_ value of the anti-DenV(E) sdAb. The experiments were repeated at least three times and representative data from one of the experiment is shown. Mean ± SEM values are shown and the levels of statistical significance was analyzed by 1-way ANOVA. ****P < 0.0001; ***P < 0.001; **P < 0.01; *P < 0.05.

Whether the anti-DenV(E) sdAb neutralize the infectivity of DenV, we infected Vero E6 cells with either the DenV or the virus pre-incubated with the anti-DenV(E) sdAb. At 3 dpi , the expression of DenV(E) protein was measured in the cell lysates by resolving the polypeptides and probing with biotinylated anti-DenV(E) sdAb. While the uninfected control cells showed no reactivity, an intense band of ∼60kDa was visible in the DenV infected cells (Fig. 2A). The Vero E6 cells added with the anti-DenV(E) sdAb neutralized DenV showed a low intensity band which suggested for the reduced infectivity of the virus in presence of the antibody (Fig. 2A). We also observed similar results when the virus infectivity was assessed by qRT-PCR wherein the transcripts of a non-structural proteins of DenV, NS3, were measured (Fig. 2B and C). Within 90 min of infection, a 4,000-fold higher expression of NS3 was detectable in the DenV infected cells as compared to those infected with the anti-DenV(E) sdAb neutralized virus (Fig. 2B). Similar results were obtained at 90 h of infection suggesting that fewer particles infected Vero cells following incubation with anti-DenV(E) sdAb which then led to reduced production of virions and consequently a lower expression of the viral gene (Fig. 2C). To further assess the neutralization of DenV by the sdAb, we performed plaque forming assays. Ten thousand pfu of DenV alone or those pre-incubated with the increasing concentrations of anti-DenV(E) sdAb were overlaid onto Vero E6 cells and the viral titers were measured 5 d later. Anti-DenV sdAb reduced the viral titers in a concentration dependent manner. We observed a 10-fold reduction in the viral titers at 40 µg concentration of the sdAb (Fig. 2D). These results showed an inhibition of the virus infectivity by anti-DenV(E) sdAb. As the anti-DenV(E) sdAb interacted with the DenV(E) protein displayed on the viral surface, we assessed its ability to interfere with the virus attachment which serves as the first step in the cellular infection process. The attachment step can be assessed by incubating the virus and the susceptible cells at 4°C as a 37°C incubation temperature could make it difficult to delineate the attachment and internalization process. To this end, we incubated 10^4^ pfu of DenV with increasing concentration of the anti-DenV(E) sdAb and the mix was then added to Vero E6 cells for 90 min kept at 4°C. The preincubation of anti-DenV(E) sdAb with the virus reduced frequencies of viral antigen positive cells when compared with the un-neutralized virus added cells, an effect that occurred in a dose dependent fashion (Fig. 2E and F). A 100 µg/ml of the sdAb reduced percent positive cells by 90% with the IC_50_ value being 1.19 µg/ml (Fig. 2E and F). Thus, anti-DenV(E) sdAb by binding to the surface displayed envelope protein inhibited the viral attachment to cell surface which rendered the internalization process inefficient and in so doing blocked viral infection.

**Figure 3.**
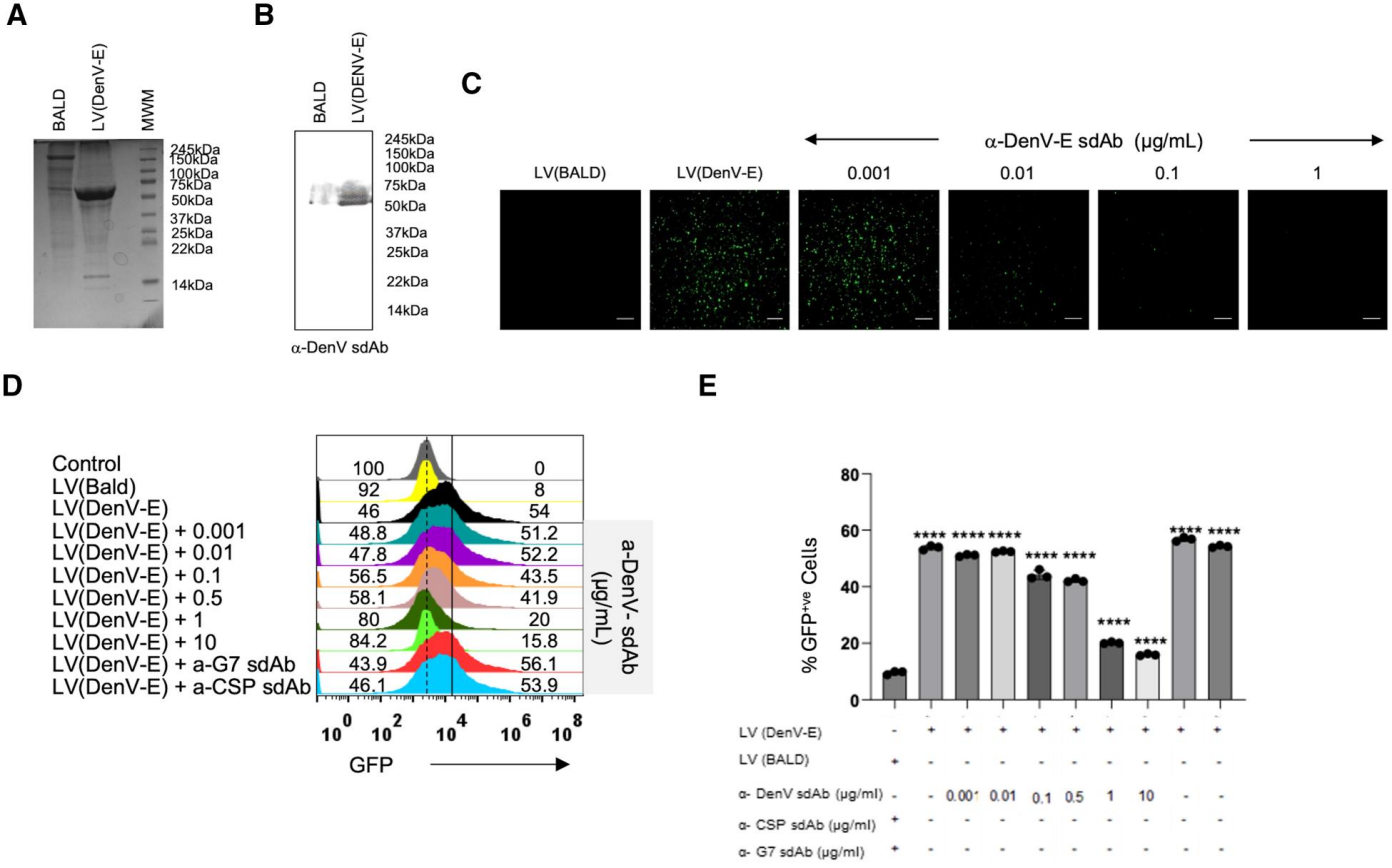
Neutralization of LV(DenV2 E) pseudoviruses by anti-DenV(E) sdAb. HEK293T cells were transfected with different plasmids as explained in materials and method to generate lentivirus based DenV(E) expressing pseudoviruses. (A) LV(DenV2 E) and LV(Bald) pseudoviruses were resolved using 12% SDS-PAGE. The expression of DenV(E) protein in the supernatant of the transfected HEK293T cells is observed as a polypeptide band of ∼55kDa. (B) Immunoblots show the expression of DenV(E) protein using anti-DenV(E) sdAb as indicated. (C) The concentrated pseudoviruses were pre-incubated with different concentration of anti-DenV(E) sdAb for 1.5 h on ice. Thereafter, the mixture was overlaid onto Vero E6 cells. After 72 h of infection cells were visualized using fluorescence microscope to measure the expression of GFP. LV(BALD) pseudoviruses serve as control. (D) Representative offset histograms show the frequencies of GFP positive cells in absence or the presence of the anti-DenV(E) sdAb using flow cytometry. (E) Bar graph showing the cumulative results as obtained in (D) The experiments were repeated at least three times and representative data from one of the experiment is shown. Mean ± SEM values are shown and the levels of statistical significance was analyzed by 1-way ANOVA. ****P < 0.0001; ***P < 0.001; **P < 0.01; *P < 0.05.

Replication competent live virus could confound the results of antibody mediated inhibition experiments as the internalization kinetics, the replication potential and the emerging mutants could result in altered fitness of the virus and affect virion production. We therefore tested the neutralizing potential of anti-DenV(E) sdAb using replication deficient pseudotyped lentivirus expressing surface DenV(E) protein. The expression of the DenV(E) protein by the LV (DenV-E) pseudovirus was confirmed through SDS-PAGE and Western blot analysis (Fig. 3A and B). We then pre-incubated different concentrations of the anti-DenV(E) sdAbs with 5 × 10^6^ LV (DenV-E) or LV(VSV-G)afterwards, this mixture was added to Vero E6 cells and the cells were analysed for GFP expression at 72 h post-infection (Fig. 3C–E). As compared to the control LV(DenV-E) pseudoviruses, those preincubated with the anti-DenV(E) sdAb showed a reduced internalisation as the concentration of anti-DenV(E) sdAb increased (Fig. 3C–E). An inhibition of 50% was achieved by 1 µg of anti-DenV(E) sdAb and a concentration of 10 µg achieved a maximum of 70% inhibition. When used as negative controls, the anti-CSP and anti-Gimap7 sdAb failed to neutralize LV(DenV-E) pseudoviruses even at high concentrations of 10 µg (Fig. 3C–E).

Therefore, the data presented here conclusively demonstrate that the anti-DenV(E) sdAb by binding to both the live replicating DenV as well as LV(DenV-E) effectively inhibited DenV infectivity in susceptible cells.

### Molecular docking and molecular dynamic simulation reveal interactions of anti-DenV(E) sdAb with EDII of DenV(E)

**Figure 4.**
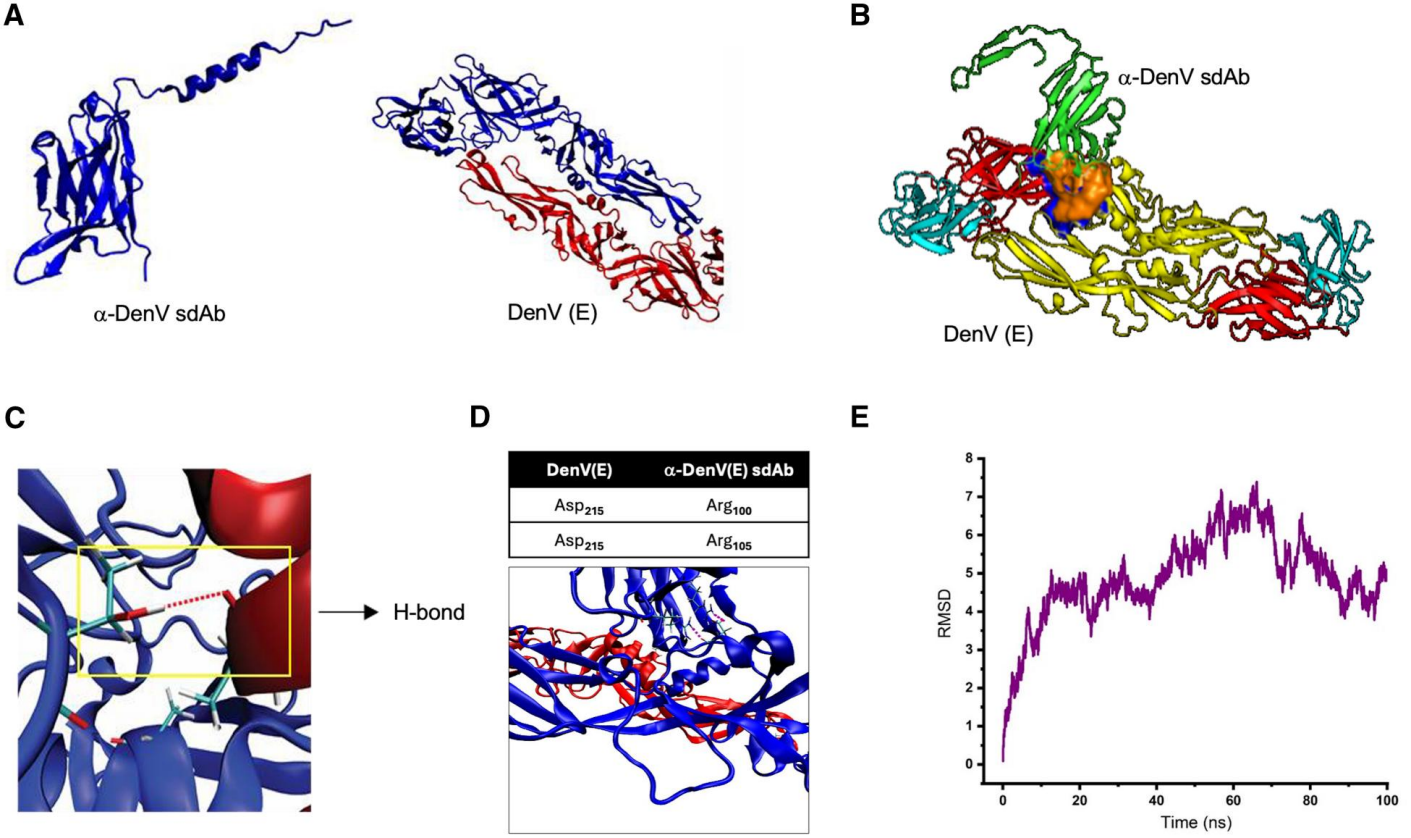
Analysing the binding of anti-DenV(E) sdAb using molecular dynamic simulation. (A) The structure of the anti-DenV(E) sdAb was predicted using Colab alphaFold (left panel) and the structure of the DenV(E) protein showing chain two chains, chain A in blue and chain B in red adapted from PDB structure (PDB_10AN) (right panel). (B) The docked model of anti-DenV(E) sdAb and DenV(E) protein shows interactions. (C) The docked model shows that an H-bond is formed between residue 76 of the anti-DenV(E) sdAb and residue 259 of the chain B of the DenV(E) protein (D) The docked model demonstrates additional interactions by two salt bridges between the anti-DenV(E) sdAb and DenV(E) protein. The interacting residues are Asp215 of chain A of DenV(E) and Arg105 and Arg100 of the anti-DenV(E) sdAb. (E) MD simulation performed for assessing the interaction of DenV(E) and anti-DenV (sdAb). Graph shows the RMSD values of interaction as molecular stimulations were analyzed for 100 ns.

The molecular docking of the amino acid sequences of the DenV(E) protein and anti-DenV(E) sdAb (Fig. 4A, right panel, Fig. S1A and B) was performed. The anti-DenV(E) sdAb interacted with DenV(E) protein via EDII. This domain is involved in the dimerization of the protein that precedes the internalization process. Therefore, the antibody might have blocked the infectivity by modulating the dimerization process of the envelope protein. Several interactions between the anti-DenV(E) sdAb and the EDII of DenV(E) protein were revealed (Fig. 4B–D). A H-bond between residue 76 of the anti-DenV(E) sdAb and residue 259 of the chain B of the DenV(E) protein was observed (Fig. 4C). Two salt bridges were also observed between the anti-DenV(E) sdAb and DenV(E) protein. Asp215 of DenV(E) interacted with Arg100 and Arg105 of the anti-DenV(E) sdAb (Fig. 4D). Both H-bond and salt-bridges could have helped stabilize the interactions between the antibody and the DenV (E) protein. We then ran a 100 ns long simulation and measured the RMSD values (Fig. 4E). These values remained constant throughout the simulation period indicating the stability of the modelled structure (Movie S1). Therefore, the molecular docking and MD simulation analysis revealed the stable interaction between the DenV(E) protein and the anti-DenV (E) sdAb.

### Anti-DenV(E) sdAb neutralizes DenV in the infected IFNRKO mice

**Figure 5.**
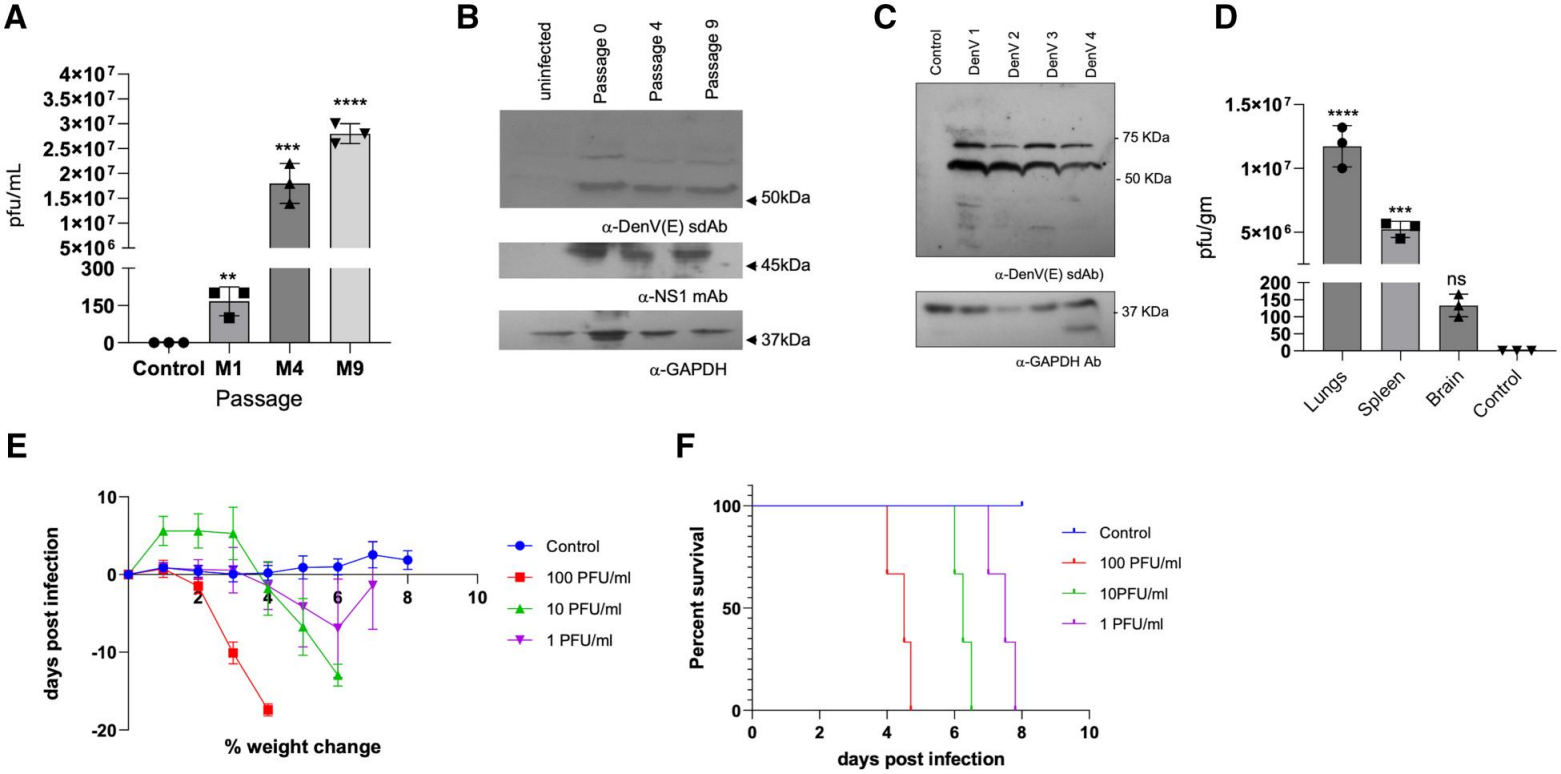
Generating a mouse adapted strain of DenV for and assessing the reactivity of anti-DenV(E) sdAb. (A) Bar graph shows the titres of DenV2 at different passages (first, fourth, and ninth) in the IFNRKO mice as measured by plaque assays using Vero E6 cells. (B) The lysates of Vero E6 cells infected with DenV obtained from 1^st^, 4^th^ and 9^th^ passage was resolved by 12%SDS-PAGE and the transferred polypeptides were detected by anti-DenV(E) sdAb. (C) Four serotypes of DenV (DenV1, 2, 3 and 4) propagated in Vero E6 cells were resolved by 12%SDS-PAGE and the transferred polypeptides were detected using anti-DenV(E) sdAb. (D) Bar graph shows the viral loads measured by plaque forming assays in the tissues of IFNRKO mice infected with DenV2. (E) Line graph depicts the % change in body weight of IFNRKO animals upon infection with different doses of DenV2 (n = 3 in each group). (F) Line graph shows the % survival of IFNRKO animals upon infection with different doses of DenV2. The experiments were repeated at least 3 times and representative data from 1 of the experiment is shown. Mean ± SEM values are shown, and the levels of statistical significance was analyzed by 1-way ANOVA. ****P < 0.0001; ***P < 0.001; **P < 0.01; *P < 0.05.

Having established the neutralization of the virus and modelling the interaction of anti-DenV(E) sdAb with the DenV (E) protein, we evaluated the virus neutralization ability of the anti-DenV(E) sdAb in vivo. To this end, we first generated a mouse adapted strain of DenV by passaging DenV2 nine times in IFNRKO mice. The supernatants of tissue lysates obtained from different organs of the infected mice in a fixed volume were injected in the subsequent passages. With every passage the viral titres increased. By the fourth and ninth passages, the viral titer increased by 10^5^ and 3 × 10^5^-fold, respectively (Fig. 5A). The accumulating mutations in the viral E protein could result in impaired binding due to altered or the lost structure recognised by the anti-DenV(E) sdAb, we therefore evaluated the ability of the anti-DenV(E) sdAb to bind to the E protein of DenV from different passages (0, 4, and 9) using western blotting. The lysates of Vero E6 cells infected with DenV from different passages were resolved and the transferred polypeptides probed with the anti-DenV(E) sdAb. The antibody detected a band of ∼60kDa corresponding to the DenV(E) protein in all the samples which suggested that the epitope recognized by anti-DenV(E) sdAb remained intact with different passages (Fig. 5B). We also evaluated the binding ability of anti-DenV(E) sdAb with the DenV (E) protein of all 4 strains of the virus grown in Vero cells and observed its efficient binding by immunoblotting (Fig. 5C). The titrated virus from passage nine was used in experiments for assessing neutralization by anti-DenV(E) sdAb in vivo. The virus propagation in different organs of IFNRKO mice was measured by collecting lungs, spleen and brain from the infected animals with an infecting dose of 1,000 pfu/mouse. The lysates from the organs were titrated using plaque assays at 3 dpi. While the virus was recovered from all the collected organs, lung tissues had the highest viral load (Fig. 5D). We then infected IFNRKO mice with different doses of DenV2 to calculate a dose which could provide us with a window for adequately monitoring the disease progression by measuring the change in body weights and the survival of infected animals. The uninfected animals gradually increased their body weights but the infected animals exhibited a significant reduction in body weights (Fig. 5E). Animals infected with 100pfu of DenV2 reduced 20% of their body weights within 4 d and all the animals in this groups eventually succumbed to the infection within five days (Fig. 5E). Inocula of 10 and 1pfu extended the survival by one and two days, respectively (Fig. 5F).

**Figure 6.**
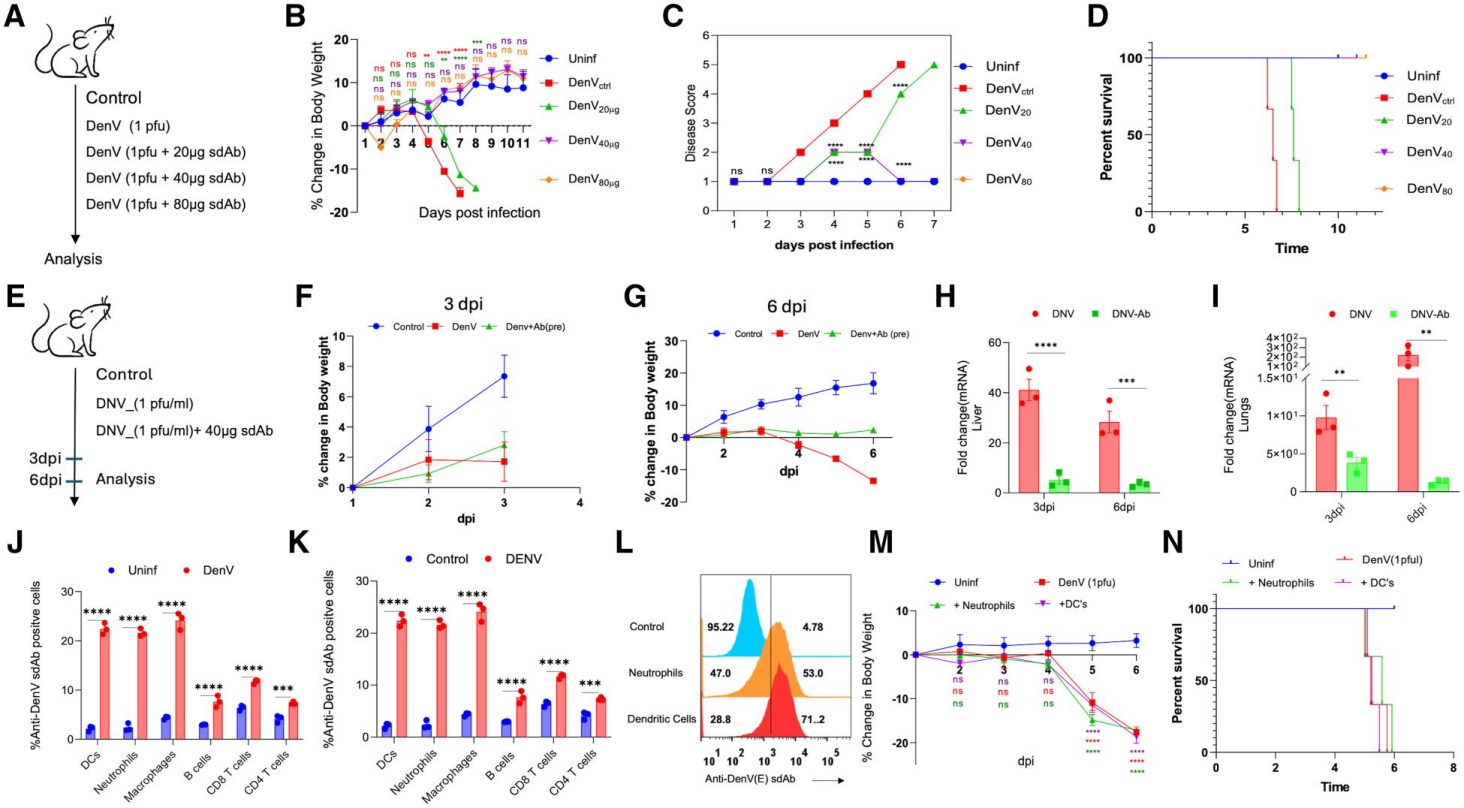
Measuring *in vivo* neutralization of a mouse adapted DenV by anti-DenV(E) sdAb in IFNRKO animals. (A) Schematic of experiments is shown. Healthy INFRKO mice of 6-8 weeks were infected with either DenV (1pfu) or the DenV (1pfu) admixed with different concentrations of anti-DenV(E) sdAb for 1.5hrs at 4°C. Subsequently, the disease score, change in bodyweight or the survival of animals in different groups of mice were recorded (n=3 in each group). (B) Line graph show the relative change in body weights of control and infected animals in different groups. (C) Line graph depicting the disease score in the control and infected animals of different groups of animals. (D) Line graphs showing the percent survival of animals in different groups (n = 3 in each group). (E) Schematic of experiments in which INFRKO mice were infected either with 1pfu of DenV, or the virus mixed with 40μg of anti-DenV sdAb at 1.5 h at 4°C. The animals were sacrificed at 3 and 6 dpi for analysing disease progress. (F, G) Line graphs show % change in body weight of IFNRKO animals upon DenV infection that were scarified at 3dpi (F) and 6dpi (G). (H–I) Bar graphs show the mRNA expression of NS3 of DenV in liver (H) and lungs (I) tissues using at 3dpi and 6dpi. The experiments were repeated at least three times and representative data from one of the experiment is shown. Mean ± SEM values are shown and the levels of statistical significance was analysed by one-way ANOVA. ****P < 0.0001; ***P < 0.001; **P < 0.01; *P < 0.05. (J-N) Expression profile of DenV in immune cells revealed innate immune cells as carrier of DenV in the IFNRKO animals. (J) Bar graph show expansion of different splenocytes in uninfected IFNRKO animals and those infected with dengue virus at 4dpi. (K) Bar graph show the expression of dengue virus E protein in different immune cells. The DenV was detected using biotinylated anti-DenV(E) sdAb followed by streptavidin-PE/APC through flow cytometry. (L) Overlaid histogram show the susceptibility of in vitro generated neutrophils and DCs from bone marrow cells for DenV. (M) Line graph showing the % change in body weight of animals infected with DenV and the recipients of adoptively transferred DenV infected neutrophils and BMDCs. (N) Line graph show percent survival of animals infected with DenV or the transferred neutrophils or BMDCs infected with DenV. The experiments were repeated at least 3 times, and data from 1 of the representative experiments is shown. Mean ± SEM values are shown and the levels of statistical significance was analysed by one-way ANOVA. ****P < 0.0001; ***P < 0.001; **P < 0.01; *P < 0.05.

We then infected IFNRKO animals with 1pfu of DenV2 for analysing the neutralizing effects of anti-DenV(E) sdAb. First, DenV2 was pre-incubated with different concentrations of anti-DenV(E) sdAb ranging from 20 µg to 80 µg for 90 min at 4°C. IFNRKO animals were infected with the control and the anti-DenV(E) sdAb preincubated virus and the animals were monitored for disease score, change in body weights and survival (Fig. 6A–D). As compared to the uninfected mice, those infected with DenV2 gradually lost body weights, developed a maximum disease score by 6 dpi (Fig. 6B and C). All the infected animals succumbed to the infection by 7 dpi (Fig. 6C). 100% of animals injected with 40 µg and 80 µg of anti-DenV (E) sdAb neutralized DenV2 survived and no significant reduction in body weights and disease symptoms were evident (Fig. 6B–D). Animals injected with 20 µg of anti-DenV(E) sdAb incubated virus showed a slower kinetics of loss in body weights and the disease development. The animals also exhibited an extended survival by a day as compared to the infected controls (Fig. 6D). The viral titres were measured in separate experiments where the animals were infected with DenV2 pre-neutralized using 40 µg of anti-DenV(E) sdAb or with the DenV2 alone (Fig. 6E). While the animals in the infected groups displayed no reduction in body weights until 3 dpi, ∼20% reduction in body weights was observed for animals in the DenV infected animals by 6 dpi (Fig. 6F and G). As compared to the infected animals, the animals injected with the pre-neutralized virus lost significantly less body weights (Fig. 6G). Liver and lung homogenates of animals given the anti-DenV(E) sdAb pre-neutralized virus had approximately 8- and 100- fold lower viral loads in comparison to those injected with the un-neutralized virus as measured by the transcripts of NS3 gene using real time qualitative polymerase chain reaction (RT-qPCR) at 6 dpi (Fig. 6H and I).

We also performed phenotypic analysis of splenocytes of IFNRKO mice infected with the DenV2 (Fig. 6J and K) at 3 dpi and observed a significant increase in the frequency of neutrophils (Gr1^+^CD11b^+^), dendritic cells (CD11c^+^CD11b^+^), macrophages (F4/80^+^CD11b^+^) and B cells (CD19^+^B220^+^) but reduction in those of CD4^+^ and CD8^+^ T cells in the DenV infected mice (Fig. 6J). Cellular tropism of DenV for different immunocytes was measured by intracellular staining using the anti-DenV(E) sdAb (Fig. 6K). While a greater fraction of the monocytic (DCs and macrophages) and granulocytic (neutrophils) cells were positive for DenV antigens, B and T cells exhibited comparatively lower positivity percentages (Fig. 6K). We, therefore, assessed whether DenV infected innate immune cells could transport virus and cause infection in the IFNRKO mice following adoptive transfer (Fig. 6L–N and Fig. S2A). Neutrophils and BMDCs generated from bone marrow precursors of IFNRKO animals were FACS-sorted and infected with DenV2 (Fig. 6L). One million of the Cell Trace Far Red^TM.^ labelled cells were then adoptively transferred in IFNRKO mice and equal transfer was established in the peripheral blood (Fig. 6L–N, Fig. S2A). The recipients were monitored for the diseases progression. The animals injected with the DenV-infected BMDCs or neutrophils rapidly lost body weights and succumb to the infection by 6 dpi as was observed for the DenV infected animals (Fig. 6L–N).

The data presented here showed that the DenV infected IFNRKO animals exhibited immune activation but succumbed to the infection by 6 dpi. The DenV-infected innate immune cells such as DCs and neutrophils could transport virus systemically. The anti-DenV(E) sdAb neutralized the infectivity of DenV and the virus thus neutralized failed to establish a patent infection in the IFNRKO animals.

### Anti-DenV(E) sdAb exhibits therapeutic value and modulates DenV induced immunopathological response

**Figure 7.**
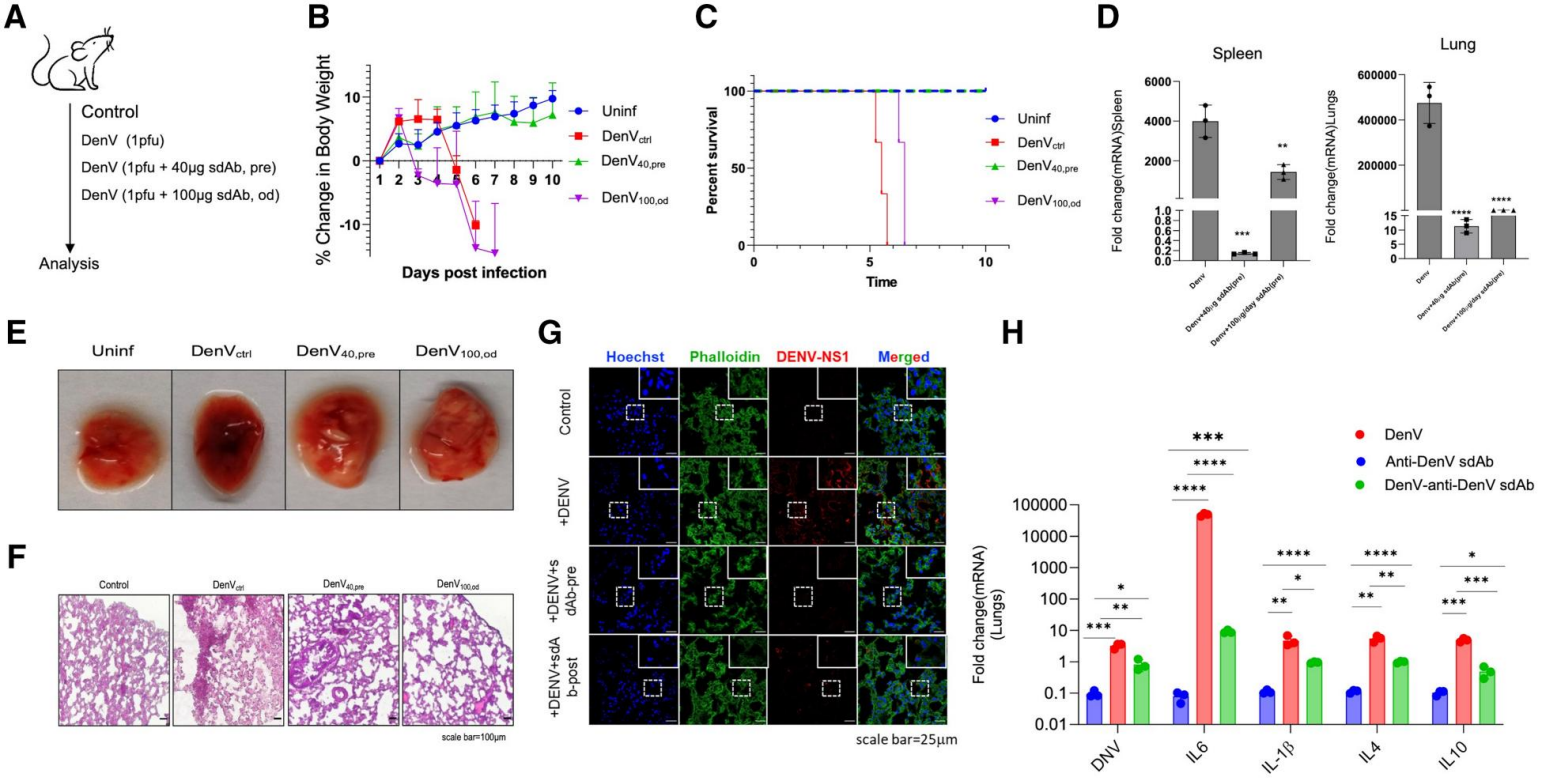
Measuring the viral load and histopathological lesions in the lungs of DenV infected IFNRKO mice injected with anti-DenV(E) sdAb. (A) Schematic of experiments is shown. INFRKO mice were infected either with 1pfu of DenV, or with a mixture of DenV and 40 μg of anti-DenV. In a separate group of animals, 100 µg of the anti-DenV(E) sdAb was injected into the animals that were infected with 1pfu of DenV one day before and the treatment was repeated until the termination of experiments (n = 3 in each group). (B) Line graph shows relative change in the body weights of the animals in different groups. (C) Line graph shows the disease score of animals in different groups. (D) Percent survival of animals is shown in different groups. (E) Representative gross images of exteriorised lungs are shown for different groups. Extensive haemorrhagic lesions are evident in animals infected with DenV while less so in those given anti-DenV (E) sdAb. (F) Bright field microscopic H and E stained sections of the lung tissues from different groups of animals were imaged. Compared with those of anti-DenV(E) sdAb injected groups, pronounced leukocytic infiltrates in the lungs of DenV infected animals are visible. (G) Confocal microscopy images of lungs tissues revealing the expression of DenV NS1 protein in the lungs of animals from the indicated groups are shown. Lungs tissues were first sectioned and then stained with nuclear stain (Hoechst), total actin (phalloidin) and anti-DenV NS1 antibody. Viral antigens were more abundant in the infected animals as compared to the additional receiving anti-DenV sdAb. (H) Indicated genes for pro- (IL-1β, IL-6) and anti- (IL-4, IL-10) inflammatory molecules as well as the gene for DenV(NS3) were amplified from the lysates of lung tissues of DenV infected animals or those infected with 100 µg of anti-DenV(E) sdAb and DenV at 5dpi. The experiments were repeated at least three times and representative data from one of the experiment is shown. Mean ± SEM values are shown and the levels of statistical significance was analysed by two- way ANOVA. ****P < 0.0001; ***P < 0.001; **P < 0.01; *P < 0.05.

To assess the therapeutic potential of anti-DenV(E) sdAb in the animals previously infected with DenV, 100 µg of the sdAb was injected 24 h post infection and the injections were repeated daily for 5 d. The body weight profile, diseases score and survival were recorded for each group (Fig. 7A). While the mice infected with DenV died on 6 dpi, the mice infected with DenV/anti-DenV(E) sdAb pre-incubated mix or the control mice (uninfected) survived till the termination of experiments (Fig. 7B and C). Mice injected with the sdAb post infection survived 24 to 36 h longer than the DenV infected mice (Fig. 7B and C). Thus, the sdAb was found effective in the virus neutralization when administered therapeutically. Lower levels of protection could be attributed to the poor retention of anti-DenV(E) sdAb owing to their smaller size which could lead to a faster renal clearance. Furthermore, even a fraction of the un-neutralized virus particles could produce more copies in the IFNRKO animals. The viral loads were measured by analysing the mRNA expression of NS3 gene by RT-qPCR. Mice from each group were sacrificed at 4 dpi to isolate lungs and spleens (Fig. 7D). The NS3 transcripts were least abundant in the anti-DenV(E) sdAb pre-incubated group and more abundant in the infected control. The tissues isolated from the DenV infected animals that were administered with the anti-DenV(E) sdAb after infection had intermediate levels of NS3 transcripts (Fig. 7D). The lung tissues of DenV infected animals showed extensive haemorrhagic lesions as compared to those injected with the anti-DenV(E) sdAb (Fig. 7E). The infected lungs showed abundant immune cell infiltration leadingd to disrupted alveolar spaces. Incontrast, the group pre-incubated with anti-DenV(E) sdAb or treated with anti-DenV(E) sdAb post infection exhibited reduced cellular infiltrates (Fig. 7F). The architecture of lung tissues in the DenV infected group was severely altered with extensive inflammatory cell infiltration and disrupted alveolar spaces, however, these signs were less pronounced in other groups (Fig. 7F). The presence of virus antigens in lung sections was examined using anti-DenV NS1 monoclonal antibody by immunofluorescence microscopy. The viral antigens were abundantly present in the DenV infected animals as compared to those additionally receiving anti-DenV(E) sdAb (Fig. 7G). Splenic and lung tissues were isolated for RNA isolation and the expression of viral DenV(NS3) genes was measured as an indicator of viral loads (Fig. 7H, Fig. S3B). The animals injected with the anti-DenV(E) sdAb showed a significant reduction in the expression of viral NS3 genes in splenic and lung tissues as compared to the control infected mice (Fig. 7H, Fig. S2B). We also measured the levels of pro- and anti-inflammatory cytokines in the lysates of lung and splenic tissues. In comparison to the DenV infected animals, those injected with anti-DenV(E) sdAb neutralized virus showed significantly less cytokine response (Fig. 7H, Fig. S2B). Interestingly, the injection of anti-DenV(E) sdAb alone did not trigger any cytokine response, a desirable attribute for a biologics used for therapy. This suggested that the antibody injection controlled the virus load more effectively which resulted in reduced inflammatory response. Therefore, the data showed that anti-DenV(E) sdAb effectively achieved *in vivo* neutralization of DenV in the infected animals and its administration provided therapeutic benefits to the infected animals.

## Discussion

Despite intense efforts spread over past three decades to mitigate the consequences of DenV infection, it continues to pose a significant health threat.^34^^,^^35^ Challenges in resolving the pathogenesis of DenV are multifaceted which include a lack of robust animal model, limited studies on the viral antigenic determinants, existence of several serotypes and the emerging genetic variants which provide limited cross protection.^36^^,^^37^ Polyclonal neutralizing antibodies are produced to target both structural (E, Pr-M, C) and non-structural (NS1, NS3, and NS5) proteins of DenV but their relative neutralization potential against heterologous serotypes has not been assessed adequately.^38^^,^^39^ Furthermore, these polyclonal antibodies could enhance the severity of re-infection especially against heterologous serotypes due to a phenomenon commonly known as ADE.^39^ The Food and Drug Administration (FDA) approved anti-DenV vaccines such as Dengvaxia and QDENGA have limited usage and are not approved for all the age groups. Other vaccines are in different stages of development. Despite these advancements, the global struggle with DenV persists.^40^^,^^41^ Monoclonal antibodies emerge as promising diagnostic and therapeutic agents for early virus detection and control, respectively. Numerous studies have identified human and murine monoclonal antibodies for DenV neutralization and some of these include B10 (targeting an E dimer epitope), mouse mAbs 3H5 (anti-DIII domain), anti-NS1 mAb, and 8H8 (anti-capsid mAb).^42–44^ However, the use of such antibodies poses a challenge due to the associated risk of ADE. Notably, the sdAbs or the nanobodies derived from the camelid *v*ariable region of the *h*eavy chain of the *h*eavy chain antibodies (V_H_H) represent a promising tool to tackle such infections as they lacks in the Fc region and thus, the risk of ADE is eliminated.

In this study, we panned out a sdAb from phage display libraries of V_H_H against the envelope protein of DenV. The DenV(E) protein is responsible for the virus entry in the susceptible cells and an antibody targeting this protein is likely to reduce the viral infectivity. The specificity of the antibody towards its cognate receptor was determined through various biophysical and immunological assays. The anti-DenV(E) sdAb neutralized pseudoviruses expressing DenV envelope protein, LV(DenV-E) as well as the virulent DenV2 *in vitro* and protected a highly susceptible IFNR KO mice from DenV infection. The antibody also enhanced the survival of infected IFNRKO mice by 24 to 36 h when the antibody therapy was initiated post infection, a particularly noteworthy outcome in the highly susceptible IFNR KO mice . The results suggest for the potential of the anti-DenV(E) sdAb in achieving better efficacy of neutralization in the immunocompetent host. However, a potential limitation could be due to their smaller size which might result in rapid renal clearance. Generating bivalent antibodies or combining the recognition module with carrier protein such as the albumin domain can efficiently help resolve this issue, a current direction of pursuit in our laboratory.^45^^,^^46^ The FDA approved sdAbs such as Caplacizumab for therapy against acquired thrombotic thrombocytopenic purpura (TTP) was modified to generate a bivalent preparation which enhanced its persistence.^47^^,^^48^

Molecular docking studies revealed an interaction between the anti-DenV(E) sdAb and the EDII domain of the DenV. EDII domain via its conserved fusion loop plays a crucial role in dimerization of the envelope protein. The inability of the envelope protein to dimerise could hinder in the viral fusion and internalization process.^49^^,^^50^ Therefore, the anti-DenV(E) sdAb could block the infectivity of DenV in the susceptible cells. Our data showed that the anti-DenV(E) sdAb exerts a potent inhibitory effect on viral entry by engaging with the DenV(E) protein. Most neutralizing antibodies target EDIII domain, a domain involved in binding to the receptors to initiate the process of internalization. Of note EDIII domain accumulates most mutations which could reduce the binding of antibodies because of the altered binding sites and consequently a poor neutralization is achieved.^51^ While the antibodies targeting EDI and EDII were shown less effective in inducing the virus neutralization, their enhanced level of cross reactivity is evident and these domains are therefore pursed with interest as therapeutic targets.^52^ The anti-DenV(E) sdAb interacted with the envelope protein of DenV2 obtained from different passages as well as it detected the protein from all four serotypes (Fig. 5B and C). These observations attest to their broad neutralizing potential and the antibody could serve as a promising therapeutic alternative for the treatment of DenV.

## Supplementary Material

vlaf012_Supplementary_Data

## Data Availability

All the data are being provided in the manuscript. The raw data would be made available upon a reasonable request to the corresponding author.
