# Supplementary material for: In vitro and in vivo neutralization of Dengue virus by a single domain antibody
Source: Immunohorizons. 2025 Apr 3;9(5):vlaf012. doi: 10.1093/immhor/vlaf012 (PMC11968175; doi:10.1093/immhor/vlaf012)
Supplement: vlaf012_Supplementary_Data [file vlaf012_supplementary_data.zip › Supp_material.pdf]

A

Dengue virus envelope gene(Predicted amino acid sequence)

>BAL05237.1 envelope protein, partial [Dengue virus 2]

MRCIGISNRDFVEGVSGGSWVDIVLEHGSCVTTMAKNKPTLDFELIKTEAKQPATLRKYCIEAKLTNTTTESRCPTQGEPSLKEEQDKRFVCKHSMVDRGWGNGCGLFGKGGIVTCAMFTCKKNM EGKIVQP
ENLEYTIVVTPHSGEEHAVGNDTGKHGKEIKVTPQSSITEAELTGYGTVTMECSPRTGLDFNEMVLLQMENRAWLVHRQWFLDLPLPWLPGADKQGSNWIQKETLVTFKNPHAKKQDVVVLGSQE GAMHTA
LTGATEIQMSSGNLLFTGHPKCRLRMDKLQLKGMSYSMCTGKFKVVKEIAETQHGTIVIRVQYEGDGSPCKIPFEIMDLEKRYVLGRLITVNPIVTEKDSPVNIEAEPPFGDSYIIIGVEPGQLKLNWFKKGSSIGQ
MFETTMRGAKRMAILGDTAWDFGSLGGVFTSIGKALHQVFGAIYGAAFSGVSWTMKILIGVIITWIGMNSRSTSLSVSLVLVGIVTLYLGVMVQA

Dengue virus envelope gene (Nucleotide sequence)

5’atgcgttgcataggaatgtcaaatagagactttgtggaagggg tttcaggaggaagctgggttgacatagtcttagaacatggaagctgtgtgacgacgatggcaaaaacaaaccaacattg gatttgaactgataaaaacagaagccaaacagcctgccaccctaaggaagtactgtat
agaggcaaagctaaccaacacaacaacagaatctcgctgcccaacacaaggggaacccagcctaaatgaagagcaggacaaaaggttcgtctgcaaacactccatggtagacagaggatggggaa atggatgtggactatttggaaagggaggcattgtgacctgtgctatgttcagatg
caaaaagaacatggaaggaaaagttgtgcaaccagaaacttggaatacaccattgtgataacacctcactcaggggaagagcatgcagtcggaaatgacacaggaaaacatggcaaggaaatcaa aataacaccacaagttccatcacagaagcagaattgacaggttatggcactgtc
acaatggagtgctctccaagaacgggcctcgacttcaatgagatggtgttgctgcagatggaaaataaagcttggctggtgcacaggcaatggttcctagacctgccgttaccatggttgcccgg agcggacacacaagggtcaaattggatacagaaagagacattggtcactttcaaaaatcc
ccatgcgaagaaacaggatgttgttgttttaggatcccaagaaggggccatgcacacagcacttacaggggccacagaaatccaaatgtcatcaggaaacttactcttcacaggacatctcaagt gcaggctgagaatggacaagctacagctcaaaggaatgtcatactctatgtgcacagg
aaagtttaaagttgtgaaggaaatagcagaaacacaacatggaacaatagttatcagagtgcaatatgaaggggacggctctccatgcaagatcccttttgagataatggatttggaaaaaagac atgtcttaggtcgcctgattacagtcaacccaattgtgacagaaaaagatagcccagtca
acatagaagcagaacctccattcggagacagctacatcatcataggagtagagccgggacaactgaagctcaactggtttaagaaaggaagttctatcggccaaatgtttgagacaacaatgagg ggggcgaagagaatggccattttaggtgacacagcctgggattttggatccttgggag
gagtgtttacatctataggaaaggctctccaccaagtctttggagcaatctatggagctgccttcagtggggtttcatggactatgaaaatcctcataggagtcattatcacatggataggaatg aa

ttcacgcagcacctcactgtctgtgacactagtattggtgggaattgtgacactgtatttgggagtcatggtgcaggcc………3’

B

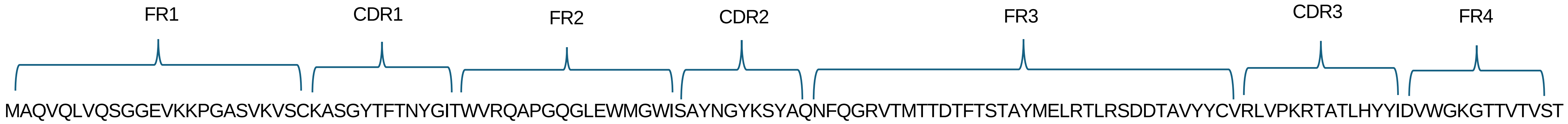

Figure S1 Sequence of the DenV (E) protein and anti-DenV(E) sdAb. A. The predicted amino acid sequence and nucleotide sequence of DenV (E) gene is shown. **B.** The predicted amino acid sequence of the anti-DenV(E) sdAb is shown wherein the framework regions (FR) are shown as FR1-4 and complementarity determining regions (CDRs) are shown as CDR1-3.

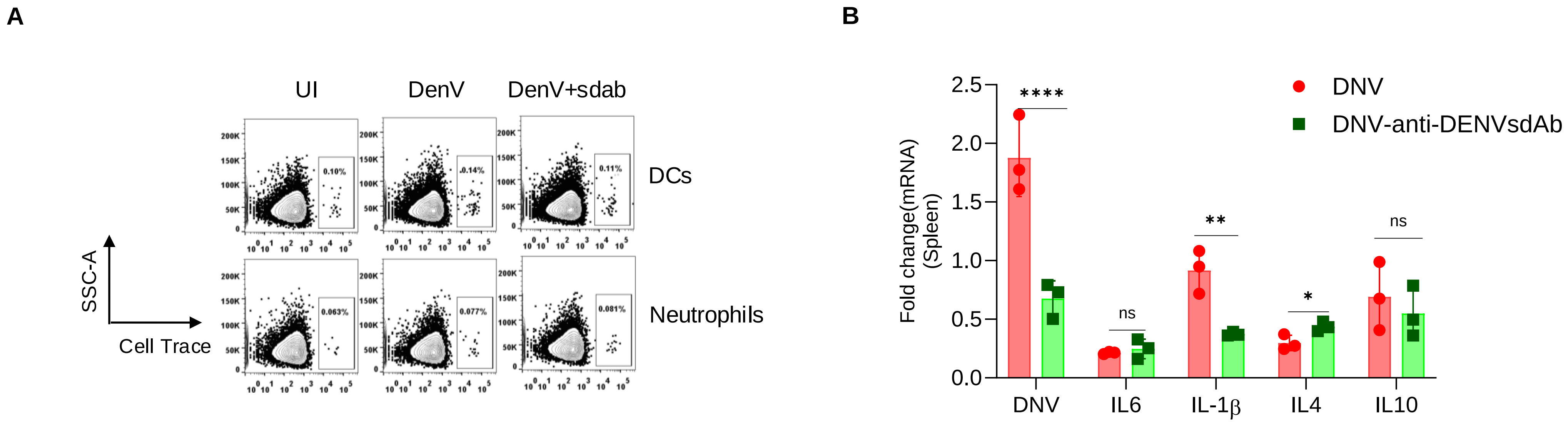

Figure S2 Assessing infectivity of DenV and the neutralization by anti-DenV(E) sdAb. A. Flow cytometry plots showing the equal adoptive transfer of *in vitro* generated DCs and neutrophils in IFNRKO mice from fig 6L. **B**. Bar diagrams show the expression of genes for pro- (*IL-1b*, *IL-6*) and anti- (*IL-4*, *IL-10*) inflammatory molecules as well as the gene for DenV(NS3) in the samples of splenic lysates from DenV infected animals or those infected with 100μg of DenV-anti-DenV(E) sdAb and DenV at 5dpi.

Movie S1: Assessing the interaction of anti-DenV(E) sdAb and DenV (E) protein using molecular dynamic simulation for 90 nanosecond.

Table S1: Sequence of the primers used.

Primer Name	Sequence
**MKSS-9 (V_HH FP)**	GTTGTGTGGAATTGTGAGCG
**MKSS-22 (V_HH RP)**	GAAATGCGGCCGCTGTGGAGACGGTGACCTG
**T7-FP**	TAATACGACTCACTATAGGGGAATTGTG
**T7-RP**	GCTAGTTATTGCTCAGCGGTGGCAGCAGC
**FP V_HH FR1 pLenti**	GCATTCTAGAGGCACCCCGGGATGGCCGATGTTCAACTGCAGGAG
**FR4 C-Myc pLenti**	GCTAGGATCCTGCAGATCCTCTTCAGAGATGAGTTTCTGCTCTGTGGAGACGGTGACCTG
**FR4 GFP_KDEL (RP)**	ATCCGTCGACGCGGCCGCTTTACAGTTCATCCTTCTTGTACAGCT
**18s rRNA (FP)**	GGCCCTGTAATTGGAATGAGTC
**18srRNA (RP)**	CCAAGATCCAACTACGAGCTT
**DenV NS3 (FP)**	TTCCATACAATGTGGCATGTCAC
**DenV NS3 (RP)**	GGAGATCCTGAGGTTCCAGGAG
**DenV2 E_FP**	ATGCGTCGACATGCGTTGCATAGGA
**DenV2 E_RP**	ATGCGCGGCCGCGGCCTGCACCATGAC
**DenV2_PMD2.G (FP)**	ATGCAAGCACGTGATGCGTTGCA
**DenV2_PMD2.G (RP)**	ATGCCTGCGGCCGCGGCCTGCAC
